# Interactive effects of atmospheric oxidative pollutants and heat on circulatory disease mortality

**DOI:** 10.3389/fpubh.2025.1629857

**Published:** 2025-07-29

**Authors:** Wenli Wang, Yang Chen, Ruoyu Li, Linsheng Yang, Yu Jiang, Jianjun Xiang, Jing Wu, Jing Li, Baoying Liu, Huaying Lin, Chuancheng Wu

**Affiliations:** ^1^Department of Preventive Medicine, School of Public Health, Fujian Medical University, Fuzhou, China; ^2^The Key Laboratory of Environment and Health, School of Public Health, Fujian Medical University, Fuzhou, China; ^3^The Affiliated Fuzhou Center for Disease Control and Prevention of Fujian Medical University, Fuzhou, China; ^4^School of Public Health, Anhui Medical University, Hefei, China

**Keywords:** oxidative stress, cardiovascular mortality, heatwaves, environmental epidemiology, lag effect, interaction analysis

## Abstract

**Background:**

Atmospheric oxidative pollutants, air temperature, and heatwave events pose potential threats to public health. However, the combined effects of these factors on the risk of mortality from circulatory disease remain insufficiently studied. This study aims to evaluate the synergistic effects of ozone (O₃), oxidant (O_x_), and nitrogen dioxide (NO₂) with temperature and heat waves, and to explore their impact on circulatory disease mortality, providing evidence to support public health interventions.

**Methods:**

Based on the mortality, meteorological, and environmental protection data of residents in Fuzhou City from January 1, 2016, to December 31, 2022, we employed a generalized additive model (GAM) and a distributed lag nonlinear model (DLNM) to assess the effects of atmospheric oxidative pollutants interacting with temperature and heat waves on the risk of death from circulatory diseases, where temperature includes the daily maximum temperature and diurnal temperature range (DTR). A bivariate three-dimensional model was used to visualize their synergistic effects, and stratified analyses were conducted to compare differences between heat wave and non-heat wave periods.

**Results:**

O_3_, O_x_, and NO_2_ exhibit synergistic effects with ambient temperature, and their combined exposure significantly increases the mortality risk of circulatory system diseases, myocardial infarction, and stroke, with some health effects showing a “nonlinear exposure-response relationship with an inverted U-shaped pattern.” Under heatwave conditions, the synergistic effect between NO_2_ and high temperatures is markedly enhanced, leading to a greater increase in mortality risk compared to O_3_ and O_x_, and demonstrating both a same-day lag and a cumulative effect. After introducing other pollutants into the dual-pollution model, NO_2_ still shows a strong independent health effect on major causes of death during heatwaves, with the most pronounced risk elevation observed for stroke.

**Conclusion:**

Atmospheric oxidative pollutants interact with high temperatures, diurnal temperature range, and heatwaves, significantly increasing the risk of mortality. It is essential to integrate air pollution and meteorological factors to strengthen health protection during high-risk periods.

## 1 Introduction

Against the backdrop of global climate change, extreme weather events are becoming increasingly frequent. At the same time, accelerated urbanization and industrialization are contributing to the gradual deterioration of air quality (1, 2). Numerous studies have confirmed that air pollution and extreme meteorological conditions can significantly increase population mortality and disease risks (3–8). Some research has also shown that high temperatures pose a risk for death from circulatory diseases in humans (9, 10). However, environmental exposure factors rarely occur in isolation. Atmospheric oxidative pollutants may interact in complex ways with meteorological variables like temperature and heatwaves, and the health impacts of such combined exposures have not been fully elucidated. Existing evidence indicates that rising temperatures can enhance ozone formation efficiency, and high heat may exacerbate the toxic effects of pollutants by altering human physiological responses (11, 12). Therefore, assessing the health effects of single exposure factors alone may underestimate the true public health risks.

As early as the 20th century, researchers began to investigate the combined effects of temperature and atmospheric oxidative pollutants (13). Subsequent studies have found that such interactions may lead to an increase in the morbidity risk in the population (14–16). Similarly, studies have indicated that the health effects of air pollution may be amplified under the combined action of meteorological fluctuations (16–18). In addition, some studies have focused on the combined effect of diurnal temperature range (DTR) and atmospheric oxidizing pollutants (19, 20). Research suggests that both extremely high and extremely low DTR may exacerbate health risks, and that very low DTR, in combination with fine particulate matter, may play a synergistic role in the development of chronic obstructive pulmonary disease (21), In addition, a review has systematically summarized the mechanisms by which air pollution, particularly PM, exacerbates conditions in chronic obstructive pulmonary disease (COPD) patients, further supporting the potential synergistic effects between pollutants and meteorological factors (22), suggesting that the combined effect of meteorological variability and air pollution deserves great attention.

Located on the southeast coast of China, Fuzhou is characterized by a hot and humid climate with intense sunlight. Although the overall air quality is better than that of northern cities, its unique climatic conditions may amplify the health impacts of high temperatures and oxidative pollutants. Therefore, conducting a population-based mortality risk assessment focused on the interactions between heatwaves, high temperatures, and atmospheric oxidative pollutants in Fuzhou could provide valuable scientific insights for other coastal cities.

## 2 Materials and methods

### 2.1 Data information

The data collected for this study consist of a continuous seven-year time series from January 1, 2016, to December 31, 2022, in Fuzhou City, including environmental protection data, meteorological data, and mortality data of residents.

#### 2.1.1 Environmental protection data

The daily average concentration data of atmospheric pollutants in this study were obtained from seven national environmental monitoring stations established by the Fuzhou Environmental Monitoring Center Station, These stations are located at Fujian Normal University, Gushan, Ziyang, Kuaian, Wusi North Road, Yangqiao West Road, and the Twenty-ninth Middle School of Fuzhou City. The pollutants monitored include PM_2.5_ (particulate matter with a diameter < 2.5 μm), PM_10_ (particulate matter with a diameter < 10 μm), O_3_, NO_2_, SO_2_ (sulphur dioxide), and CO (carbon monoxide).

Atmospheric oxidizing capacity is used to assess the ability of atmospheric oxidants to oxidize or reduce substances. Referring to the relevant literature (23), O_x_ can be estimated by the hourly mass concentrations (hereinafter referred to as concentrations) of O_3_ and NO_2_. Due to the different redox potentials of O_3_ and NO_2_, different weights are assigned to the two when calculating O_x_ in this study. The formula for calculating O_x_ is as follows (Equation 1):


(1) $$\rho_{\mathrm{ox}}=[(1.070\times \rho_{\mathrm{NO}2})+(2.075\times \rho_{O3})]/3.145$$

#### 2.1.2 Meteorological data

Meteorological data were obtained from the Meteorological Bureau of Fuzhou City, including daily average temperature (°C), daily average atmospheric pressure (hPa), and daily average relative humidity (%).

#### 2.1.3 Mortality data

Mortality data in this study were obtained from the Fuzhou Center for Disease Control and Prevention, based on joint reporting from public security, civil affairs, and other relevant departments. Daily death counts were classified according to the underlying cause of death based on the “International Classification of Diseases, the 10th Edition (ICD-10).” The categories included deaths from circulatory system diseases (I00–I99), myocardial infarction (I21), and stroke (I64).

### 2.2 Statistical analysis

This study employed a bivariate three-dimensional model and stratified analysis to assess the interactive effects of atmospheric oxidative pollutants and temperature on mortality risk among residents. The bivariate three-dimensional model was used to visualize the joint effects of oxidative pollutants and temperature, providing an intuitive representation of their relationship with mortality risk. Stratified analysis was conducted to evaluate the effects of pollutants separately during heatwave and non-heatwave periods, to explore variations under different meteorological conditions.

#### 2.2.1 Bivariate three-dimensional model of oxidative pollutants and heatwaves

In this study, thin plate regression splines (TPS) were first used to visualize the interactive effects of temperature and atmospheric oxidative pollutants on mortality risk. Subsequently, temperature and atmospheric oxidative pollutants were included as continuous bivariate predictors in a GAM to generate a three-dimensional surface plot illustrating their relationships with the outcome variable. Considering the lagged effect of atmospheric oxidative pollutants on health outcomes, the pollutant concentrations in the model used the moving average with a lag of L days (lag0-L), where the value of L was determined based on the lag effect analysis. The model formula is as follows:


$$\begin{array}{l} \log(E(Y_{t}))=\mathrm{TS}(P_{\mathrm{tL}},{\text{Temp}}_{t})+\mathrm{ns}(\text{Time},\mathrm{df})+\\ \mathrm{ns}(X_{t},\mathrm{df})+\mathrm{Dow}+\text{holiday}+\alpha \end{array}$$

In this equation, TS represents TPS, TS(P_tL_, Temp_t_) represents the interaction term between atmospheric oxidizing pollutants and temperature, P_tL_ is the moving average of atmospheric oxidizing pollutant day L on day t, Temp_t_ is the daily average temperature on day t, and others are as in Equation 1.

#### 2.2.2 Interaction model of oxidizing pollutants and heatwaves

This study focused exclusively on periods during which heatwaves were likely to occur, limiting the analysis to the summer months (May to October) from 2016 to 2022. According to the World Meteorological Organization (WMO) definition, a heatwave is identified when the daily maximum temperature exceeds 32°C for three consecutive days (24). To examine the interactive effect between atmospheric oxidizing pollutants and heatwaves, we employed a GAM. The effects of oxidative pollutants on mortality risk are assessed separately during heatwave and non-heatwave periods. The model also adjusted for long-term time trends, short-term fluctuations, and potential confounding effects of other meteorological factors. The model formula is as follows:


$$\begin{array}{l} \log(E(Y_{t}))=\beta_{K}Z_{t}H_{K}+\mathrm{ns}(\text{Time},\mathrm{df})+\\ \mathrm{ns}(X_{t},\mathrm{df})+\mathrm{Dow}+\text{holiday}+\alpha \end{array}$$

In this equation, E (Y_t_) is the expected value of the number of deaths on day t, Z_t_ is the oxidizing pollutant on day t, β is the exposure-response coefficient, H_K_ denotes a dummy variable for heatwave (H_1_ = 0, H_2_ = 0) and non-heatwave days (H_1_ = 0, H_2_ = 0), β_1_ and β_2_ represent the effects of oxidizing pollutants in the heatwave layer and the non-heatwave layer respectively, X_t_ is the meteorological factor on day t of the observation, including the average relative humidity, and others as in Equation 1.

### 2.3 Quality control

This study employed dedicated data management personnel responsible for the collection and quality control of mortality, environmental, and meteorological monitoring data. The personnel were required to be familiar with the data collection content and related workflows, including data acquisition, quality review, and online submission. All collected data had to be accurate, with complete geographic coverage and comprehensive variables. In particular, key variables such as date of death, cause of death, and ICD codes were required to be fully documented. Rigorous verification of reported data was conducted to eliminate duplicate, erroneous, or redundant records, as well as data from days with abnormally high or low death counts, to ensure data quality and the accuracy of the analysis.

## 3 Results

### 3.1 Bivariate model results for the effects of O_3_, O_x_, NO_2_ and temperature on mortality

#### 3.1.1 Bivariate model results for the effects of O_3_, O_x_, NO_2_ and daily maximum temperature on mortality

**Figure 1:**
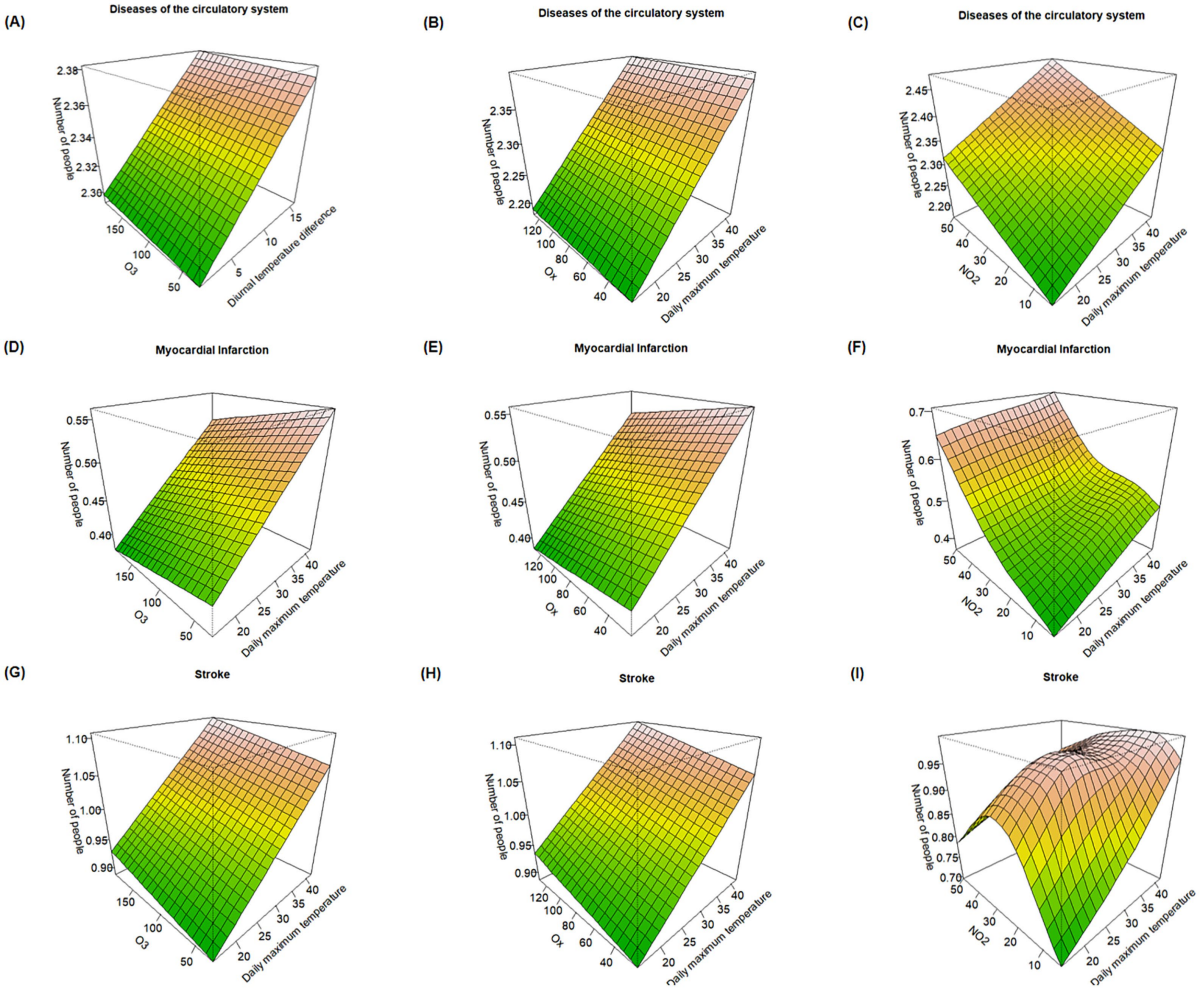
Response surface plots of the effects of O_3_, O_x_, and NO_2_ combined with daily maximum temperature on major causes of death. **(A–C)** Effect of O_3_, O_x_, and NO_2_ combined with daily maximum temperature on mortality risk from circulatory system diseases. **(D–F)** Effect of O_3_, O_x_, and NO_2_ combined with daily maximum temperature on mortality risk from myocardial infarction. **(G–I)** Effect of O_3_, O_x_, and NO_2_ combined with daily maximum temperature on mortality risk from stroke.

Visualization of O_3_, O_x_, NO_2_, and May–October daily maximum temperature with the risk of death from circulatory system diseases, myocardial infarction, and stroke (Figure 1), the results showed that increases in O_3_ and O_x_ concentrations, along with rising daily maximum temperatures, were associated with an upward trend in mortality risk for all three causes of death. This suggests that the combined exposure to daily maximum temperature and oxidative pollutants may have a greater impact on mortality than the independent effects of each factor alone. Further observation revealed that deaths from circulatory system diseases and myocardial infarction increased markedly with rising NO_2_ concentrations and daily maximum temperatures, with the mortality effects peaking when both NO_2_ levels and daily maximum temperatures were at their highest. These findings indicate a possible synergistic effect between NO_2_ and high temperatures, posing a heightened health threat for specific diseases.

In addition, the interactive effects between atmospheric oxidative pollutants and daily maximum temperature exhibited differentiated health impacts across various causes of death. While the effects of O_3_ and O_x_ on mortality from circulatory system diseases, myocardial infarction, and stroke followed similar overall trends, the dominant influencing factors varied. For circulatory system and stroke mortality, daily maximum temperature was the primary driver. In contrast, NO_2_ showed a significant leading effect on myocardial infarction mortality, with risk increasing as NO_2_ concentration rose. Notably, the interaction effect between NO_2_ and daily maximum temperature presents some health effects showing a “nonlinear exposure-response relationship with an inverted U-shaped pattern.” That is to say, when the concentration of NO_2_ approaches 30 μg/m^3^, the combined effect with high temperature is the strongest, and it has the greatest impact on the mortality risk of stroke among residents.

#### 3.1.2 Bivariate model results for the effects of O_3_, O_x_, NO_2_ and DTR on mortality

**Figure 2:**
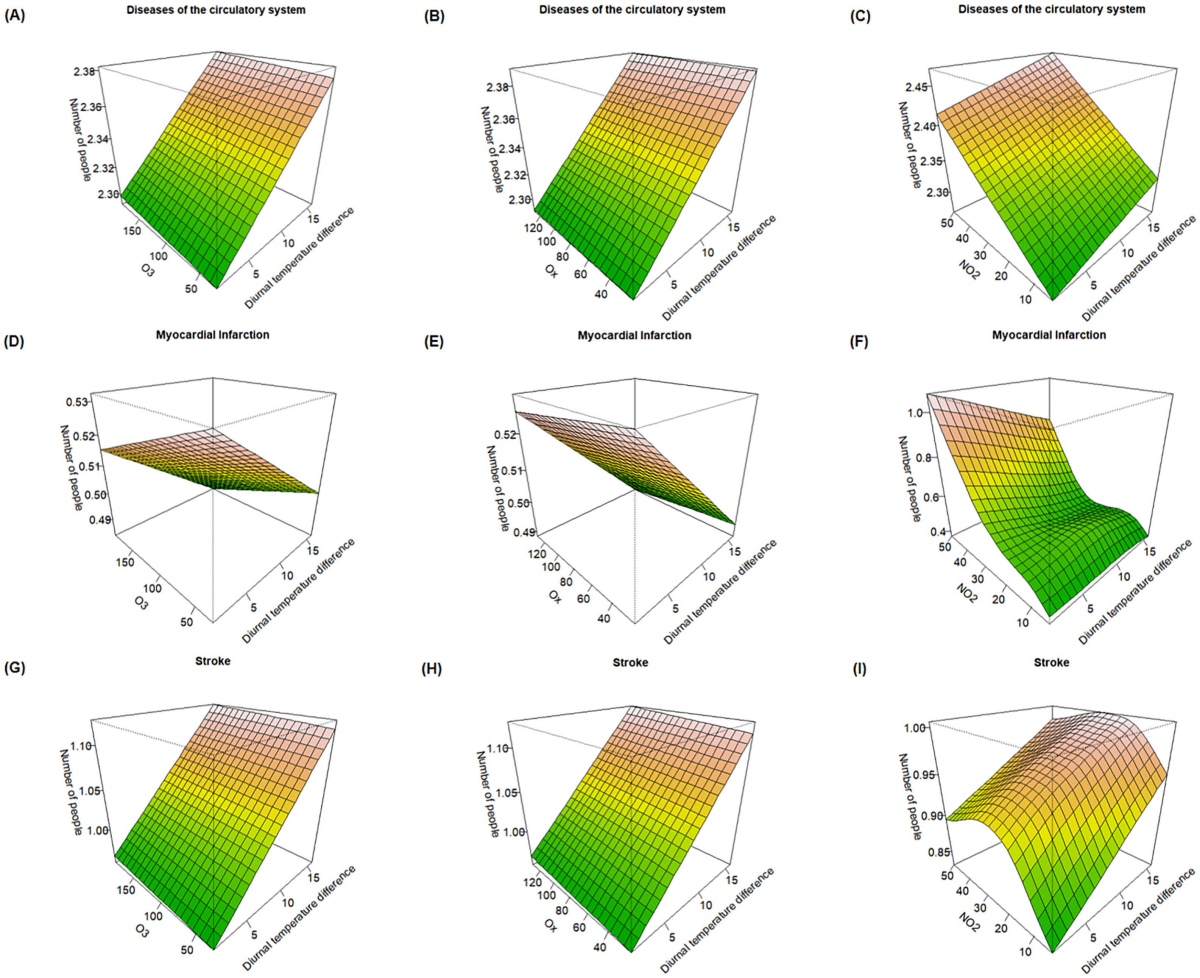
Response surface plots of the effects of O_3_, O_x_, and NO_2_ combined with DTR on major causes of death. **(A–C)** Effect of O_3_, O_x_, and NO_2_ combined with DTR on mortality risk from circulatory system diseases. **(D–F)** Effect of O_3_, O_x_, and NO_2_ combined with DTR on mortality risk from myocardial infarction. **(G–I)** Effect of O_3_, O_x_, and NO_2_ combined with DTR on mortality risk from stroke.

Figure 2 presents bivariate three-dimensional surface plots illustrating the effects of O_3_, O_x_, NO_2_, and DTR from May to October on deaths due to circulatory system diseases, myocardial infarction, and stroke. The plots show that when O_3_ and O_x_ concentrations, as well as DTR, reached their highest levels, deaths from circulatory system diseases and stroke also peaked. This suggests that the combined effect of DTR with O_3_ and O_x_ may exceed their individual impacts in triggering mortality from these two conditions. Additionally, when both NO_2_ concentration and DTR increased, the response surface for circulatory system disease mortality exhibited an upward trend, indicating that their joint effect may elevate the mortality risk. As for myocardial infarction, the combined effects of O_3_, O_x_, and NO_2_ with DTR showed that mortality risk generally rose with increasing pollutant concentrations. However, interestingly, higher DTR was associated with fewer deaths, suggesting a potential inverse relationship in this context.

The interactions between different atmospheric oxidative pollutants and DTR demonstrated varying effects across different diseases. For mortality from circulatory system diseases and stroke, DTR appeared to be the dominant factor in the interaction effects. In contrast, for myocardial infarction, O_3_ and O_x_ played the primary roles, with risk decreasing as the concentrations of O_3_ and O_x_ increased. In the interaction between NO_2_ and DTR, NO_2_ concentration emerged as the dominant factor influencing circulatory system diseases and myocardial infarction death. Additionally, the interaction effect between NO_2_ and DTR varied in awith some health effects showing a “nonlinear exposure-response relationship with an inverted U-shaped pattern,” with the largest effect of the interaction with DTR at about 30 μg/m^3^, which corresponded to the highest number of stroke deaths in the population.

### 3.2 Interaction effects of atmospheric oxidizing pollutants and heatwaves on residential mortality

#### 3.2.1 Results of the bivariate model of the effects of atmospheric oxidizing pollutants and heatwaves on residential mortality

**Figure 3:**
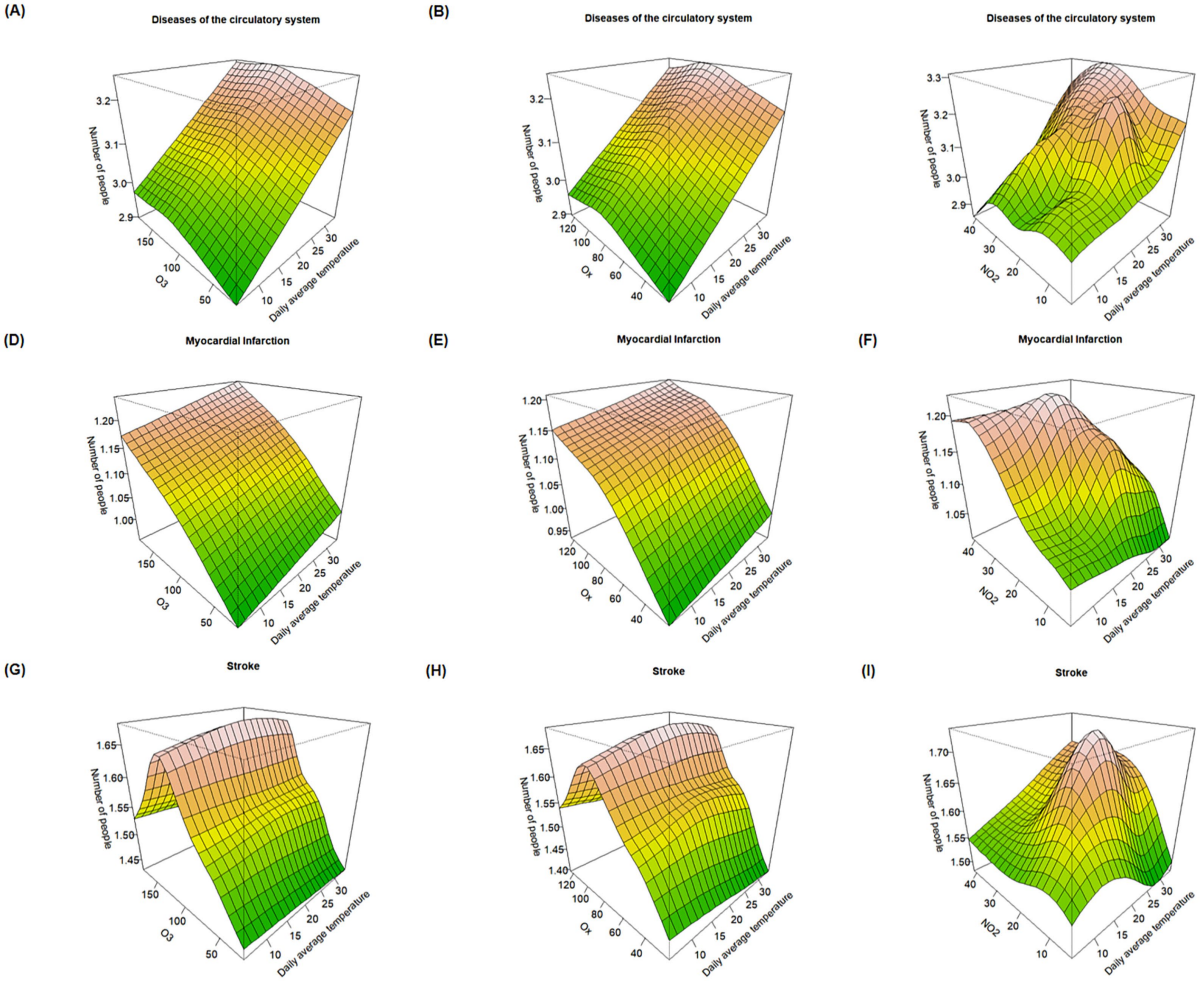
Response surface plots of the effects of O_3_, O_x_, and NO_2_ combined with temperature on major causes of death during heatwave periods. **(A–C)** Effect of O_3_, O_x_, and NO_2_ combined with temperature on mortality risk from circulatory system diseases. **(D–F)** Effect of O_3_, O_x_, and NO_2_ combined with temperature on mortality risk from myocardial infarction. **(G–I)** Effect of O_3_, O_x_, and NO_2_ combined with temperature on mortality risk from stroke.

As shown in Figure 3, under heatwave temperature conditions, the joint effects of O_3_ and O_x_ concentrations with temperature on circulatory system disease risk follow a similar upward trend as observed under general daily maximum temperature conditions. However, for stroke mortality, the risk appears to vary only with changes in O_3_ and O_x_ concentrations, suggesting that under heatwave conditions, the interaction between temperature and these pollutants no longer significantly influences stroke mortality. Moreover, in the interaction between O_3_, O_x_, and temperature on circulatory system disease mortality, temperature emerges as the dominant contributing factor.

When both NO_2_ concentration and temperature increase simultaneously, the number of deaths from circulatory system diseases, myocardial infarction, and stroke among residents shows an upward trend. This suggests that under heatwave conditions, the combined effect of NO_2_ and temperature on mortality may exceed the effects of either factor alone.

**Figure 4:**
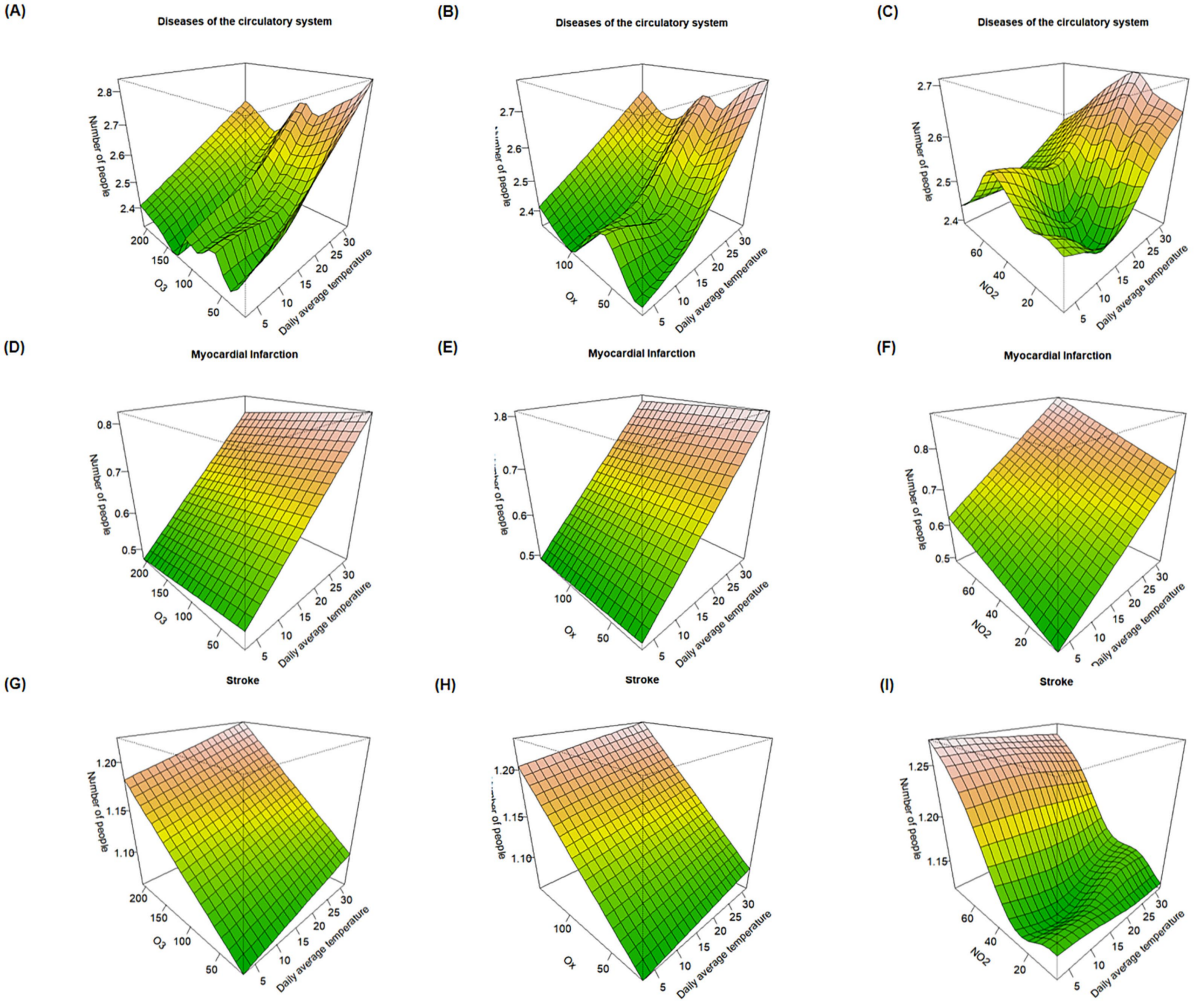
Response surface plots of the effects of O_3_, O_x_, and NO_2_ combined with temperature on major causes of death during non-heatwave periods. **(A–C)** Effect of O_3_, O_x_, and NO_2_ combined with temperature on mortality risk from circulatory system diseases. **(D–F)** Effect of O_3_, O_x_, and NO_2_ combined with temperature on mortality risk from myocardial infarction. **(G–I)** Effect of O_3_, O_x_, and NO_2_ combined with temperature on mortality risk from stroke.

Compared to heatwave days, under non-heatwave temperature conditions (as shown in Figure 4), the mortality effect of stroke reaches its peak when both O_3_ or O_x_ concentrations and temperature are at their highest, implying that on non-heatwave days, the combined impact of temperature and O_3_ and O_x_ may exert a stronger influence on stroke mortality. Overall, however, the mortality effects observed during heatwaves are higher than those on non-heatwave days, suggesting that heatwave events can exacerbate the effects of oxidizing pollutants on the risk of residential mortality.

#### 3.2.2 Effects of the interaction between atmospheric oxidative pollutants and heatwaves on the excess mortality of major causes of death

**Figure 5:**
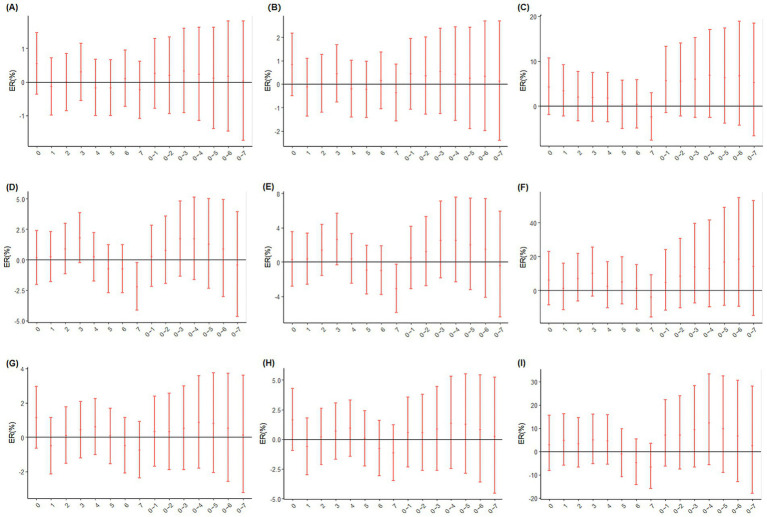
Excess risk for major causes of death associated with each 10 μg/m^3^ increase in O_3_, O_x_, and NO_2_ concentrations during heatwave days (ER: Excess Risk and 95% CI: 95% Confidence Interval). **(A–C)** Effect of each 10 μg/m^3^ increase in O_3_, O_x_, and NO_2_ on the risk of circulatory system disease mortality among residents. **(D–F)** Effect of each 10 μg/m^3^ increase in O_3_, O_x_, and NO_2_ on the risk of myocardial infarction mortality among residents. **(G–I)** Effect of each 10 μg/m^3^ increase in O_3_, O_x_, and NO_2_ on the risk of stroke mortality among residents.

During heatwaves, each 10 μg/m^3^ increase in concentrations of O_3_, O_x_, and NO_2_ was associated with both a lagged effect of the day (lag0) and a persistent cumulative effect (lag0–7) excess mortality effects on circulatory system diseases, myocardial infarction, and stroke, as shown in Figure 5. Further analysis of the lag0 day effect revealed that the health risks associated with NO_2_ during heatwave periods were the most pronounced, exceeding those of O_3_ and O_x_. Table 1 also presents a comparison between the results of this study and those reported in previous literature, suggesting that NO_2_ may exhibit a stronger independent health effect under high-temperature conditions. To assess the robustness of the heatwave definition, a sensitivity analysis using an alternative threshold of 32°C lasting for two consecutive days was performed, and the results are presented in Supplementary Table S1. The findings were consistent with the main analysis, indicating the stability of the observed effects.

**Table 1:** Stratified analysis of the impact of each 10 μg/m^3^ increase in O_3_, O_x_, and NO_2_ on excess risk among residents, with the heatwave definition of ≥32°C for ≥3 consecutive days (ER and 95% CI).

Pollutant	Heatwave category	Circulatory system diseases	Myocardial infarction	Stroke	ER (95% CI) - Reference	References
O_3_	Heatwave days	0.55 (−0.37, 1.47)	0.15 (−2.02, 2.37)	1.14 (−0.64, 2.95)	0.31 (0.25–0.37)	(41)
	Non-heatwave days	−0.23 (−0.94, 0.49)	0.26 (−1.41, 1.97)	0.75 (−0.55, 2.06)		
O_x_	Heatwave days	0.83 (0–0.50, 2.17)	0.30 (−2.83, 3.54)	1.63 (−0.94, 4.28)	None available	
	Non-heatwave days	−0.19 (−1.26, 0.90)	0.58 (−1.96, 3.18)	1.44 (−0.53, 3.45)		
NO_2_	Heatwave days	4.22 (−1.91, 10.74)	6.00 (−8.50, 22.81)	2.96 (−8.23, 15.50)	1.04 (1.02–1.05)	(42)
	Non-heatwave days	1.36 (−0.48, 3.24)	1.47 (−2.79, 5.91)	2.46 (−0.80, 5.83)		

### 3.3 Fitting results of the two-pollutant model for the interaction between atmospheric oxidative pollutants and heatwaves

Based on the lag day with the highest excess risk (ER), additional pollutants were introduced to construct two-pollutant models. The results showed that, after adjusting for PM_2.5_, PM_10_, SO_2_, and CO during non-heatwave periods, the effects of atmospheric oxidative pollutants on the mortality risks of circulatory system diseases, myocardial infarction, and stroke were not statistically significant (*p* > 0.05). However, during heatwave periods, the single-pollutant models for O_3_ and O_x_ indicated a positive association with increased risk of myocardial infarction mortality, with ER values (95% CI) of 0.15 (−2.02, 2.37) and 0.30 (−2.83, 3.54), respectively. After incorporating PM_2.5_, PM_10_, SO_2_, and CO into the two-pollutant models, the effects of O_3_ and O_x_ on myocardial infarction mortality were attenuated. This attenuation may be attributed to reduced outdoor activity during extreme heat, thus reducing the actual exposure to air pollutants.

In addition, within the two-pollutant models for NO_2_, its effects on mortality from circulatory system diseases, myocardial infarction, and stroke were significantly stronger than those of O_3_ and O_x_. In particular, the ER (95% CI) of the effect of the two-pollutant model of NO_2_ and PM_10_ on stroke deaths was 4.84 (−12.00, 24.90), with a peak value nearly 8 times greater than that of O_3_ (2.29) or O_x_ (3.54). Detailed results are presented in Tables 2–4.

**Table 2:** Two-pollutant model for the effect of each 10 μg/m^3^ increase in O_3_ on the excess mortality risk of the major causes of death among residents (ER and 95% CI).

Model		Heatwave days	Non-heatwave days
Circulatory system diseases	O_3_	0.55 (−0.37, 1.47)	−0.23 (−0.94, 0.49)
	O_3_ + PM_2.5_	−0.29 (−1.54, 0.97)	−0.26 (−0.99, 0.48)
	O_3_ + PM_10_	−0.43 (−1.74, 0.91)	−0.28 (−1.00, 0.46)
	O_3_ + NO_2_	0.29 (−0.80, 1.39)	−0.18 (−0.89, 0.54)
	O_3_ + SO_2_	0.45 (−0.55, 1.46)	−0.27 (−0.99, 0.46)
	O_3_ + CO	0.11 (−1.03, 1.26)	−0.24 (−0.95, 0.48)
Myocardial infarction	O_3_	0.15 (−2.02, 2.37)	0.26 (−1.41, 1.97)
	O_3_ + PM_2.5_	−0.32 (−3.34, 2.78)	0.15 (−1.58, 1.92)
	O_3_ + PM_10_	−0.98 (−4.15, 2.29)	0.25 (−1.46, 1.99)
	O_3_ + NO_2_	−0.51 (−3.14, 2.19)	0.33 (−1.36, 2.04)
	O_3_ + SO_2_	−0.07 (−2.47, 2.39)	0.11 (−1.59, 1.83)
	O_3_ + CO	−1.53 (−4.22, 1.24)	0.11 (−1.57, 1.81)
Stroke	O_3_	1.14 (−0.64, 2.95)	0.75 (−0.55, 2.06)
	O_3_ + PM_2.5_	1.21 (−1.24, 3.73)	0.60 (−0.73, 1.96)
	O_3_ + PM_10_	2.16 (−0.44, 4.84)	0.57 (−0.75, 1.90)
	O_3_ + NO_2_	1.28 (−0.85, 3.45)	0.86 (−0.44, 2.18)
	O_3_ + SO_2_	1.28 (−0.67, 3.26)	0.73 (−0.58, 2.06)
	O_3_ + CO	1.67 (−0.56, 3.96)	0.75 (−0.55, 2.06)

**Table 3:** Two-pollutant model for the effect of each 10 μg/m^3^ increase in O_x_ on the excess mortality risk of the major causes of death among residents (ER and 95% CI).

Model		Heatwave days	Non-heatwave days
Circulatory system diseases	O_x_	0.83 (−0.50, 2.17)	−0.19 (−1.26, 0.90)^*^
	O_x_ + PM_2.5_	−0.43 (−2.28, 1.45)	−0.24 (−1.39, 0.92)
	O_x_ + PM_10_	−0.68 (−2.65, 1.34)	−0.30 (−1.43, 0.84)
	O_x_ + NO_2_	0.44 (−1.21, 2.12)	−0.27 (−1.35, 0.82)
	O_x_ + SO_2_	0.70 (−0.77, 2.19)	−0.29 (−1.40, 0.84)
	O_x_ + CO	0.19 (−1.48, 1.89)	−0.21 (−1.31, 0.89)
Myocardial infarction	O_x_	0.30 (−2.83, 3.54)	0.58 (−1.96, 3.18)
	O_x_ + PM_2.5_	−0.34 (−4.77, 4.29)	0.37 (−2.34, 3.15)
	O_x_ + PM_10_	−1.41 (−6.12, 3.54)	0.58 (−2.10, 3.32)
	O_x_ + NO_2_	−0.77 (−4.72, 3.33)	0.50 (−2.05, 3.11)
	O_x_ + SO_2_	−0.02 (−3.52, 3.61)	0.21 (−2.42, 2.90)
	O_x_ + CO	−2.20 (−6.10, 1.86)	0.11 (−2.45, 2.74)
Stroke	O_x_	1.63 (−0.94, 4.28)	1.44 (−0.53, 3.45)
	O_x_ + PM_2.5_	1.77 (−1.88, 5.55)	1.21 (−0.89, 3.35)
	O_x_ + PM_10_	3.36 (−0.60, 7.48)	1.05 (−1.01, 3.15)^*^
	O_x_ + NO_2_	1.94 (−1.28, 5.27)	1.31 (−0.67, 3.32)^*^
	O_x_ + SO_2_	1.87 (−0.99, 4.81)	1.46 (−0.58, 3.54)^*^
	O_x_ + CO	2.47 (−0.83, 5.87)	1.47 (−0.53, 3.51)^*^

*p < 0.01 indicates a statistically significant difference between heatwave and non-heatwave days.

**Table 4:** Two-pollutant model for the effect of each 10 μg/m^3^ increase in NO_2_ on the excess mortality risk of the major causes of death among residents (ER and 95% CI).

Model		Heatwave days	Non-heatwave days
Circulatory system diseases	NO_2_	4.22 (−1.91, 10.74)	1.36 (−0.48, 3.24)
	NO_2_ + PM_2.5_	−0.18 (−7.68, 7.93)	1.74 (−0.39, 3.92)
	NO_2_ + PM_10_	−2.17 (−10.74, 7.22)	1.64 (−0.62, 3.96)
	NO_2_ + O_3_	3.12 (−4.12, 10.91)	1.32 (−0.54, 3.21)
	NO_2_ + SO_2_	3.89 (−3.55, 11.91)	1.47 (−0.66, 3.64)
	NO_2_ + CO	1.77 (−5.42, 9.50)	1.58 (−0.47, 3.69)
Myocardial infarction	NO_2_	6.00 (−8.50, 22.81)	1.47 (−2.79, 5.91)
	NO_2_ + PM_2.5_	6.73 (−11.78, 29.11)	1.15 (−3.71, 6.25)^*^
	NO_2_ + PM_10_	3.99 (−16.98, 30.26)	1.93 (−3.28, 7.41)
	NO_2_ + O_3_	8.10 (−9.62, 29.30)	1.56 (−2.73, 6.03)^*^
	NO_2_ + SO_2_	6.12 (−11.48, 27.22)	0.33 (−4.49, 5.40)^*^
	NO_2_ + CO	−1.52 (−17.52, 17.59)	−0.61 (−5.22, 4.23)
Stroke	NO_2_	2.96 (−8.23, 15.50)	2.46 (−0.80, 5.83)
	NO_2_ + PM_2.5_	−0.10 (−13.86, 15.85)	2.07 (−1.66, 5.94)
	NO_2_ + PM_10_	4.84 (−12.00, 24.90)	1.40 (−2.50, 5.46)^*^
	NO_2_ + O_3_	−1.60 (−14.28, 12.97)	2.70 (−0.59, 6.10)
	NO_2_ + SO_2_	3.46 (−10.19, 19.18)	2.97 (−0.82, 6.90)
	NO_2_ + CO	3.61 (−9.81, 19.03)	2.98 (−0.68, 6.78)

*p < 0.01 indicates a statistically significant difference between heatwave and non-heatwave days.

## 4 Discussion

### 4.1 Synergistic effects of O_3_, O_x_, and temperature on circulatory system mortality

This study found that simultaneous increases in daily maximum temperature and O_3_ concentrations were significantly associated with elevated mortality from circulatory system diseases, myocardial infarction, and stroke. These findings suggest a potential synergistic health effect between O_3_ and high temperatures, which is consistent with previous research. For example, one study reported that on high-ozone days, a 1°C increase in temperature was associated with a 2.20% rise in daily total mortality (25). In addition, diurnal temperature range (DTR), an indicator of intraday temperature fluctuation, was incorporated into our analysis to assess its joint effects with O_3_ and O_x_. The results indicated that these interactive effects were particularly pronounced in stroke-related mortality. While limited epidemiological evidence currently exists regarding the interaction between DTR and O_3_, our study provides novel insights into this area.

From a biological perspective, O_3_ may exacerbate cardiovascular burden through multiple mechanisms, including the activation of oxidative stress pathways, promotion of systemic inflammation, induction of myocardial ischemia, and impairment of endothelial function (26, 27). These mechanisms provide a plausible explanation for the amplified health risks observed under conditions of high temperature or significant temperature variability.

Regarding O_x_, which reflects the combined oxidant burden of ozone and NO_2_ (28), our findings demonstrated that elevated O_x_ levels in conjunction with high temperature and DTR significantly increased the risk of circulatory system and stroke-related mortality. This may be attributed to the toxicological effects of NO_x_ compounds (29). Notably, most previous studies have focused on the relationship between NO_x_ and daily mean temperature (30, 31). In contrast, this study introduces DTR as an indicator of temperature variability and reveals that its interaction with O_x_ has a more pronounced impact on health under thermally unstable conditions. This finding adds methodological innovation and real-world significance to the current literature.

### 4.2 The impact of O_3_ and O_x_ in combination with heatwaves on circulatory mortality

During heatwave periods, the synergistic effects between elevated concentrations of O_3_, O_x_, and high ambient temperatures further amplified the mortality risks from circulatory system diseases and myocardial infarction, suggesting a compounded health burden driven by their interaction. This finding is consistent with previous research (32–34). Studies have indicated that under extreme heat conditions, individuals experience rapid fluctuations in heart rate, blood pressure, and blood volume, which heighten their susceptibility to air pollutants (35). Moreover, heatwaves are often accompanied by intense solar radiation that promotes photochemical reactions, thereby increasing ambient O_3_ levels and further exacerbating health risks (34).

Notably, our study also observed differential effects of O_3_ and O_x_ on various causes of death. The impact was particularly significant for myocardial infarction, while the effect on stroke appeared weaker. These differences may be attributed to variations in disease pathophysiology, organ-specific susceptibility, and the timing of exposure windows. Further analysis revealed that extreme weather events may shift the dominant drivers of health risk. During non-heatwave periods, temperature emerged as the primary determinant, whereas during heatwaves, pollutant levels became the leading contributors to mortality. This dynamic shift in the primary influencing factor highlights the complex coupling between meteorological variables and air pollutants under extreme climate conditions. It underscores the need to incorporate these interactions into future climate adaptation strategies and public health early warning systems.

### 4.3 Synergistic effects of NO_2_ with temperature and its independent contribution

NO_2_ exhibited more pronounced health effects than O_3_ and O_x_ in this study, especially under high-temperature conditions and when the DTR was large. Under elevated temperatures, pollutants like NO_2_ can more easily penetrate the respiratory system and enter the bloodstream, triggering systemic inflammation and endothelial dysfunction (36). Additionally, NO_2_ may activate the sympathetic nervous system, potentially leading to arrhythmias or acute cardiovascular events. Moreover, the interaction between NO_2_ and large DTR suggests that sharp intra-day temperature swings can weaken physiologic adaptation to environmental stressors, thereby magnifying the toxic effect of NO_2_ (37, 38). Importantly, two-pollutant models showed that the NO_2_-related risks remained significant after adjusting for CO, PM_2.5_, and O_3_, indicating that NO_2_ acts as an independent hazard rather than merely serving as a proxy for overall traffic-related pollution. This result provides empirical support for prioritizing NO_2_ monitoring and targeted interventions in refined pollution-control strategies. To design still more accurate public-health measures, future studies should elucidate the molecular pathways that underlie the interaction between NO_2_ and various temperature metrics.

### 4.4 Amplified interaction between NO_2_ and heatwaves on circulatory mortality

During heatwaves, the amplifying effect of NO_2_ on the mortality risk of myocardial infarction was significantly enhanced, suggesting a heightened health sensitivity under extreme weather conditions. Our study found that the NO_2_-related risk of myocardial infarction mortality was markedly higher on heatwave days compared to non-heatwave periods. Notably, this association remained statistically significant in two-pollutant models after controlling for other co-pollutants, indicating that the observed effect may not simply reflect confounding by heat or coexisting pollutants but may instead point to an independent health effect of NO_2_ itself. In contrast, during non-heatwave periods, although the ER estimate for myocardial infarction in the NO_2_ and O_3_ two-pollutant model was the highest (ER = 8.10, 95% CI: −9.62, 29.30), it was not statistically significant—potentially due to limited sample size—highlighting the need for larger-scale studies to confirm the consistency and robustness of this trend.

From a behavioral exposure perspective, heat waves often lead to increased window ventilation and outdoor activity, unintentionally elevating actual NO_2_ exposure. On the atmospheric chemistry level, high temperatures and ultraviolet radiation accelerate NO_2_ photolysis and promote ozone formation (39, 40). As such, NO_2_ acts both as a precursor to and a product of ozone, engaging in complex nonlinear interactions that intensify under high-temperature conditions—thereby amplifying the health burden of combined pollution exposure.

In the context of increasingly frequent heatwaves, special attention should be paid to the joint exposure risks of NO_2_ and high temperatures, and enhanced control strategies are urgently needed. We recommend multi-tiered interventions during heatwaves, such as restricting high-emission vehicles, optimizing urban traffic flows, improving air purification in public spaces, and strengthening public guidance on protective behaviors, to effectively mitigate the cardiovascular risks arising from the interaction between NO_2_ and extreme heat.

### 4.5 Strengths and limitations of the study

This study presents several strengths and limitations, offering valuable insights for future research and methodological improvements. First, it is based on 7 years of comprehensive mortality data from the general population in Fuzhou and employs both GAM and DLNM to systematically evaluate the nonlinear and lagged effects of air pollutants and meteorological factors, enabling a more robust analysis. Second, by incorporating the interaction between O_x_ and DTR under a subtropical coastal climate, the study expands the regional evidence base for the “heat–oxidant synergy” hypothesis on circulatory system health. Third, the use of two-pollutant models provides further validation of the independent health effect of NO_2_, offering scientific support for refining pollutant-specific regulatory strategies and developing targeted public health interventions during heatwaves.

However, some limitations should be acknowledged. Firstly, several ER estimates had wide confidence intervals, and certain results did not reach statistical significance, possibly due to limited sample size. Future studies with larger datasets are needed to confirm the stability of these findings. Secondly, exposure assessment relied on average concentrations from fixed monitoring stations, which may not accurately capture individual-level exposure, especially during heatwave conditions when indoor–outdoor temperature differences may be substantial. Additionally, the models did not account for potential confounding factors such as socioeconomic status, pre-existing health conditions, or individual behavior, which may have influenced the exposure–response relationships. Future research could benefit from incorporating high-resolution exposure data and individual-level health records to enhance analytical precision.

## 5 Conclusion

This study, drawing on city-wide mortality records for Fuzhou from 2016 to 2022, systematically evaluated how atmospheric oxidative pollutants (O_3_, O_x_ and NO_2_) interact with meteorological factors—including daily maximum temperature, heat-waves and DTR—to influence deaths from circulatory system diseases, myocardial infarction and stroke. We found pronounced synergistic effects between O_3_, O_x,_ and both high temperature and DTR: combined exposure produced substantially higher health risks than either pollutant or weather variable alone, and the interaction patterns as well as the dominant driving factors differed by pollutant. O_3_ and O_x_ were most strongly linked to stroke mortality under large DTR, suggesting that sharp within-day temperature swings may impair physiological adaptation and amplify oxidative-stress pathways. By contrast, NO_2_ showed the greatest effect modification under high-temperature and heat-wave conditions, with a particularly strong impact on myocardial-infarction mortality. Two-pollutant models confirmed NO_2_’s independent health effect; its risk estimates remained significant after adjusting for other pollutants, indicating that NO_2_ is not merely a proxy for traffic emissions but likely exerts direct toxic effects.

Moreover, the impact of NO_2_ during heat waves was markedly stronger than on non-heat-wave days, underscoring heightened exposure sensitivity during extreme weather and NO_2_’s elevated cardiovascular hazard under high-temperature scenarios. The photochemical production and transformation of O_3_ and NO_2_ in hot conditions create complex, nonlinear pollutant–weather interactions that further intensify population-level health risks.

This study enhances our understanding that pollutants and climatic factors do not act in isolation on human health but often interact to produce compounded adverse effects. Therefore, strategies aimed at preventing and controlling the health hazards of air pollution must also consider the synergistic impact of adverse weather conditions, particularly in the context of climate change and the increasing frequency of extreme heat events.

## Data Availability

The raw data supporting the conclusions of this article will be made available by the authors, without undue reservation.
