# Supplementary material for: Interactive effects of atmospheric oxidative pollutants and heat on circulatory disease mortality
Source: Front Public Health. 2025 Jul 29;13:1629857. doi: 10.3389/fpubh.2025.1629857 (PMC12339441; doi:10.3389/fpubh.2025.1629857)
Supplement: Supplementary file 1 [file Table_1.DOCX]

Supplementary Material

# Supplementary Tables

**Supplementary Table S1.** Sensitivity analysis of the association between a 10μg/m^3^ increase in O_3_, O_x_, and NO_2_ and excess mortality risk during heatwaves defined as ≥32°C for ≥3 consecutive days (ER and 95% *CI*)

|  |  | Circulatory System Diseases | Myocardial Infarction | Stroke |
| --- | --- | --- | --- | --- |
| O_3_ | Heatwave Days | 0.68(-0.28,1.65) | 0.30 (-1.95,2.58) | 1.26 (-0.58,3.02) |
|  | Non-Heatwave Days | -0.25 (-0.95,0.45) | 0.24 (-1.45,1.92) | 0.71 (-0.52,2.00) |
| O_x_ | Heatwave Days | 0.95 (-0.42,2.34) | 0.42 (-2.73,3.68) | 1.72 (-0.89,4.33) |
|  | Non-Heatwave Days | -0.22 (-1.28,0.86) | 0.55 (-1.92,3.13) | 1.40 (-0.51,3.39) |
| NO_2_ | Heatwave Days | 4.40 (-1.78,10.85) | 6.15 (-8.30,22.90) | 3.01 (-8.10,15.60) |
|  | Non-Heatwave Days | 1.33 (-0.50,3.20) | 1.45 (-2.75,5.85) | 2.42 (-0.75,5.80) |
